# Anæsthesia—The Law

**Published:** 1881-01

**Authors:** 


### ANÆSTHESIA—THE LAW.

The administration of anæsthetics, in the absence of all parties except the patient and operator has so frequently been attended with unpleasant occurrences, that the attention of law makers has been often called to the subject; and the result has been that in Ohio the following law has been enacted.

The law is a just one, and should be scrupulously obeyed. The penalty is not severe, but is, perhaps, sufficiently so to prevent violation of the law. Every dentist who administers, or attempts to administer, a general anæsthetic of any kind, should fully understand this law.

" SEC. 6990. Whoever uses upon another an anæsthetic, unless at its administration, and during the whole time the person is wholly or partly under the direct influence of it, there is present a third person, competent to be a witness, shall be fined not more than twenty-five, nor less than five dollars."

